# Long-Term Trends in Antimicrobial Resistance Among Gram-Negative Clinical Isolates at Mubarak Al-Kabeer Hospital, Kuwait (2007–2022)

**DOI:** 10.3390/antibiotics15050501

**Published:** 2026-05-17

**Authors:** Amani H. Al-Fadhli, Ahmad Al-Dhumair, Jenan AlShemerri, Fatema Al-Failakawy, Mohammad Al-Hasan, Qadreyah Ahmad Almatawah, Wafaa Y. Jamal

**Affiliations:** 1 Laboratory Sciences, Department of Medical, College of Allied Health Sciences, Kuwait University, Jabriya 24923, Kuwait; 2 Food Security Program, Kuwait Institute for Scientific Research, Safat, Kuwait City 13109, Kuwait; aazmi@kisr.edu.kw (A.A.-D.); mhasan@kisr.edu.kw (M.A.-H.); qmutawa@kisr.edu.kw (Q.A.A.); 3 Barts and the London School of Medicine and Dentistry, Queen Mary University of London, London E1 2AT, UK; j.n.alshemeri@smd23.qmul.ac.uk; 4 Department of Laboratory Medicine, Mubarak Al Kabeer Hospital, Hawally 32052, Kuwait; q8queen_79@hotmail.com; 5 Department of Microbiology, College of Medicine, Kuwait University, Jabriya 46300, Kuwait

**Keywords:** antibiotic resistance, Kuwait, Gram-negative bacteria, surveillance, trends

## Abstract

Objectives: To examine the long-term trends in antimicrobial resistance (AMR) among major Gram-negative pathogens including *Escherichia coli*, *Klebsiella pneumoniae*, *Acinetobacter baumannii* and *Pseudomonas aeruginosa* collected from inpatient and outpatient specimens in Mubarak Al-Kabeer Hospital in Kuwait from 2007 to 2022. Methods: The antimicrobial resistance data for 39,200 non-duplicate Gram-negative isolates were collected from the Hospital Laboratory Information System (LIS). Retrospectively antibiotic susceptibility data were interpreted according to Clinical and Laboratory Standards Institute (CLSI) breakpoints with intermediate results classified as resistant. Logistic regression was applied to assess temporal trends in resistance for the following antibiotic cefotaxime, ceftazidime, ciprofloxacin, meropenem and imipenem. False discovery rate (FDR) correction was performed for multiple comparisons. Results: Third-generation cephalosporin resistance increased significantly, from 27% to 60% in *Klebsiella pneumoniae* and from 19% to 45% in *Escherichia coli*. Resistance to ciprofloxacin also increased, from 22% to 49% in *K. pneumoniae* and from 28% to 41% in *E. coli*. Notably, meropenem resistance in *K. pneumoniae* increased from 1% to 35% during the study period. *Acinetobacter baumannii* showed high resistance to most antibiotics (>75%), while colistin retained good activity (<2% resistance). By contrast, *Pseudomonas aeruginosa* showed relatively stable resistance patterns with only modest changes in susceptibility to key antibiotics. Conclusions: From 2007 to 2022, increasing resistance among major Gram-negative pathogens was observed, with cefotaxime resistance rising from 27% in 2007 to 60% in 2022 in *Klebsiella pneumoniae* and from 19% to 45% in *Escherichia coli*. Resistance to ciprofloxacin also increased over time. These findings highlight the increasing burden of antimicrobial resistance over time and emphasize the need for continued surveillance.

## 1. Introduction

Antimicrobial resistance (AMR) is globally recognized as a threat to human health [1]. The World Health Organization (WHO) has categorized AMR as one of the top 10 public health threats to humans [2]. An estimation of 4.95 million deaths was associated with AMR infections worldwide in 2019 alone [1]. Predictions are even more alarming, as the WHO reported that by 2050, deaths could reach 10 million [3]. In response to these high prediction numbers, the WHO initiated the Global Antimicrobial Resistance Surveillance System (GLASS) to monitor AMR trends [4,5]. By 2021, 70 countries had shared data on over 3 million infections with GLASS, demonstrating the global surveillance effort to control this issue [5].

Gram-negative pathogens, including *Escherichia coli*, *Klebsiella pneumoniae*, *Enterobacter aerogenes*, and *Acinetobacter baumannii*, are major causes of life-threatening infections, including bloodstream infections, urinary tract infections, and hospital-acquired pneumonia [6]. These pathogens can rapidly acquire resistance and become non-susceptible to broad-spectrum cephalosporins and carbapenems, which are considered last-resort therapies [7,8,9]. Notably, several of these organisms are part of the ESKAPEE group of pathogens, which are recognized globally for their ability to “escape” the effects of antimicrobial agents and are considered priority targets for surveillance and control efforts. These pathogens are associated with high morbidity, mortality, and healthcare burden due to their multidrug-resistant profiles [1].

Accordingly, the WHO has listed carbapenem-resistant *Acinetobacter*, *Pseudomonas*, and *Enterobacterales*, including *E. coli*, *K. pneumoniae*, as the highest-priority pathogens [10,11].

Even with worldwide attention to AMR, significant geographical gaps remain in the antibiotic resistance surveillance database. Publications on AMR trend data are relatively limited in the Middle East and North Africa (MENA) region, particularly in the Gulf states [12]. The lack of long-term surveillance data has hindered understanding of local resistance patterns and delayed the development of region-specific treatment guidelines. However, available studies showed a worryingly high prevalence of resistance among Gram-negative pathogens in Kuwait and the Gulf region [13,14]. A recent review from Kuwait reported that 77% of *E. coli* clinical isolates and 36% of *K. pneumoniae* isolates were resistant to major antibiotics, reflecting high resistance levels consistent with global reports of increasing AMR [14]. Another report documented high resistance rates in pathogens such as *P. aeruginosa* (over 90%) and *A. baumanni* (over 80%) in Saudi Arabian hospitals [14]. Recent studies from the Kingdom of Bahrain have reported that *Klebsiella pneumoniae* isolates exhibit extensive phenotypic and genotypic antimicrobial resistance, including resistance to nearly all commonly prescribed antibiotics [15]. Whole-genome sequencing (WGS) data from the Kingdom of Bahrain revealed important genomic insights and molecular epidemiological characteristics of clinical *Serratia marcescens* ST367 isolates, including the dissemination of antimicrobial resistance-associated determinants among clinically relevant strains [16]. These reported patterns highlighted the urgent need for comprehensive, local AMR surveillance to inform infection control and antibiotic stewardship in the region.

Tertiary care settings provide valuable data for long-term surveillance of antimicrobial resistance; however, such data should be interpreted with caution, particularly in the absence of stratification by infection type, patient setting, and specimen source, as well as antimicrobial consumption data (e.g., defined daily dose [DDD] or defined daily dose per 1000 inhabitants per day [DHD]). This study aimed to assess long-term trends in antimicrobial resistance among Gram-negative clinical isolates at Mubarak Al-Kabeer Hospital, Kuwait, over 16 years (2007–2022), and to characterize resistance patterns among major Gram-negative pathogens, as well as temporal changes in susceptibility to commonly used antimicrobial agents.

## 2. Results

The following sections present descriptive trends in antimicrobial resistance over time for major Gram-negative pathogens, based on isolate–antibiotic susceptibility test results.

### 2.1. Study Population and Isolate Distribution

Antibiotic susceptibility data were collected from over 16 years (2007–2022), with data available for nine reporting years (2007, 2008, 2012, 2013, and annually from 2018 to 2022). The absence of data for certain years (2009–2011 and 2014–2017) reflects gaps in electronic data availability and archival limitations within the laboratory information system during earlier periods, rather than a true absence of isolates. These gaps were considered when interpreting temporal trends.

After elimination of duplicate isolates, defined as repeated isolates of the same species from the same patient and specimen source within 30 days, a total of 39,200 non-duplicate Gram-negative isolates were included. Only the first isolate was retained for analysis. Moreover, not all isolates were tested against all antimicrobial agents; therefore, the number of observations varies by antibiotic, and analyses were performed using isolate–antibiotic susceptibility test results as the unit of analysis. For some antimicrobial agents, susceptibility data were not available for the entire study period due to changes in testing practices or the introduction of specific antibiotics during later years. Therefore, resistance trends are presented for the periods in which data were available and should be interpreted accordingly.

*Escherichia coli* (43.8%; 17,173/39,200), *Klebsiella pneumoniae* (21.5%; 8439/39,200), *Pseudomonas aeruginosa* (14.4%; 5643/39,200), and *Acinetobacter baumannii* (9.1%; 3557/39,200) were the most predominant organisms isolated. Collectively, these organisms accounted for the majority of isolates. The remaining 10% included *Enterobacter* spp., *Proteus* spp., *Citrobacter* spp., *Salmonella* spp., and *Haemophilus influenzae* (Supplementary Tables S1–S4). In addition, an overview of resistance patterns across all species and antibiotics is presented in Supplementary Figure S1. Subsequent sections provide detailed results for each organism. Overall, resistance trends from 2007 to 2022 showed a general increase in resistance to third-generation cephalosporins and fluoroquinolones in *E. coli* and *K. pneumoniae*. In contrast, *A. baumannii* remained consistently highly resistant across most antibiotic classes, while Pseudomonas aeruginosa demonstrated relatively stable resistance patterns with only modest changes over time.

### 2.2. Antimicrobial Resistance Trends in Klebsiella pneumoniae

**Figure 1:**
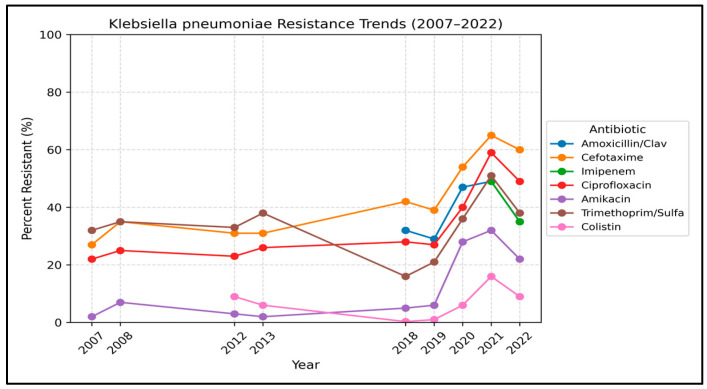
Annual resistance trends in *Klebsiella pneumoniae* (2007–2022). Percent resistant over time for key antibiotics. Dashed segments indicate years with missing data.

The trends of resistance of *K. pneumoniae* are shown in Table 1 and illustrated in Figure 1. Carbapenem resistance increased significantly over time. A total of 8439 non-duplicate *K. pneumoniae* were included in this section. Meropenem resistance increased from 1% in 2008 to 35% in 2022 (OR 1.62 per year, *p* < 0.001). Approximately one-third of *Klebsiella* isolates were carbapenem-resistant, underscoring the growing concern about carbapenem-resistant *Klebsiella*.

As with carbapenem resistance, resistance to third-generation cephalosporins increased significantly. Cefotaxime resistance increased by more than double from 27% in 2007 to 60% in 2022, and ceftazidime increased from 27% to 58% over the same period (OR 1.11 per year, *p* < 0.001 for both). Moreover, resistance to cefuroxime and cefoxitin (tested in 2007–2022) increased significantly (cefuroxime 29% to 64% and cefoxitin 12% to 45%, *p* < 0.001 for both).

In contrast, resistance to nitrofurantoin (a drug used for urinary tract infections) dropped significantly from 75% in 2007 to 40% in 2022 (OR 0.86 per year, *p* < 0.001).

Piperacillin was tested in 2021 and showed a significant decline in resistance (52% to 42%, OR < 1.0, *p* < 0.001). In contrast, piperacillin-tazobactam resistance in *K. pneumoniae* (used 2007–2008, then used from 2018 onward) was approximately 40%.

*K. pneumoniae* resistance to fluoroquinolones increased significantly. Ciprofloxacin resistance increased from 22% in 2007 to 49% in 2022 (OR 1.11 per year, *p* < 0.001). Similarly, amikacin resistance increased from 2% in 2007 to 22% in 2022 (OR 1.25, *p* < 0.001). In contrast, resistance to amoxicillin-clavulanate stayed at the same level (34%).

### 2.3. Antimicrobial Resistance Trends in Acinetobacter baumannii

**Figure 2:**
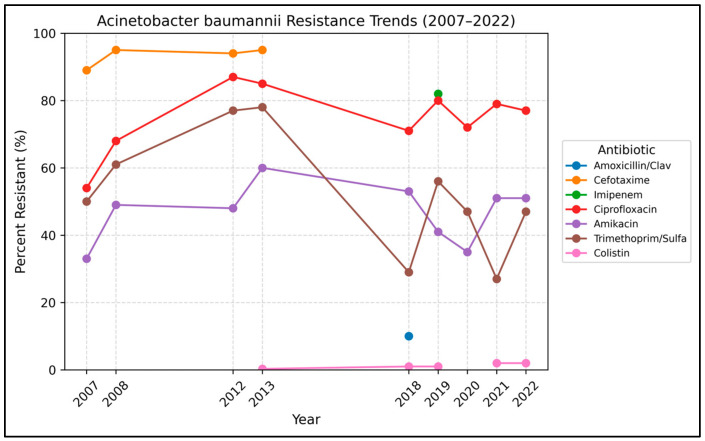
Annual resistance trends in *Acinetobacter baumannii* (2007–2022). Percent resistant over time for key antibiotics. Dashed segments indicate years with missing data.

Trends in *Acinetobacter baumannii* resistance are summarized in Table 2 and Figure 2. A total of 3557 non-duplicate *A. baumannii* isolates were analyzed and reported. Starting in 2007, *A. baumannii* showed a high resistance rate to multiple antibiotics, with over 50% of isolates resistant to meropenem, imipenem and ciprofloxacin. Over time, the resistance rate to amikacin, piperacillin-tazobactam, tigecycline, and cefotaxime (see Table 2) continued to increase.

*A. baumannii* resistance to meropenem increased significantly from 33% in 2007 to 77% in 2022 (OR 1.07 per year, *p* < 0.001). In addition, imipenem resistance ranged from 85% in 2012 to 78% in 2020 (OR 0.93, *p* < 0.001), with a slight downward trend. Overall, more than three-quarters of the reported isolates showed resistance to carbapenems.

Similar to carbapenem, resistance to piperacillin-tazobactam increased significantly after its introduction into routine testing (not tested prior to 2018) from 5% in 2018 to 78% in 2022 (OR 2.31 per year, *p* < 0.001). In contrast, piperacillin, tested from 2007 until discontinued in 2018, showed a significant downward trend in resistance over time, from 59% in 2007 to 7% in 2018 (OR 0.69, *p* < 0.001).

Colistin remained one of the few active antibiotics against *A. baumannii* in the reporting isolates, yet a slight increase in colistin resistance was observed, from 0.3% in 2013 to 2.0% in 2022 (*p* = 0.014). Overall, resistance to colistin remains very low (<2%), although the upward trend is concerning given colistin’s role as last-line therapy.

Ampicillin was introduced and tested from 2007 to 2013. During its use, the resistance increased from 90% to 100% (*p* < 0.001), approaching resistance by 2013. This demonstrates that all reported *A. baumannii* isolates were resistant to ampicillin.

By 2022, *A. baumannii* isolates were reported to have very high resistance rates to most antibiotic classes. More than 75% of reported isolates were resistant to key agents, including cefotaxime, piperacillin–tazobactam, imipenem, meropenem, and ciprofloxacin.

### 2.4. Antimicrobial Resistance Trends in Escherichia coli

**Figure 3:**
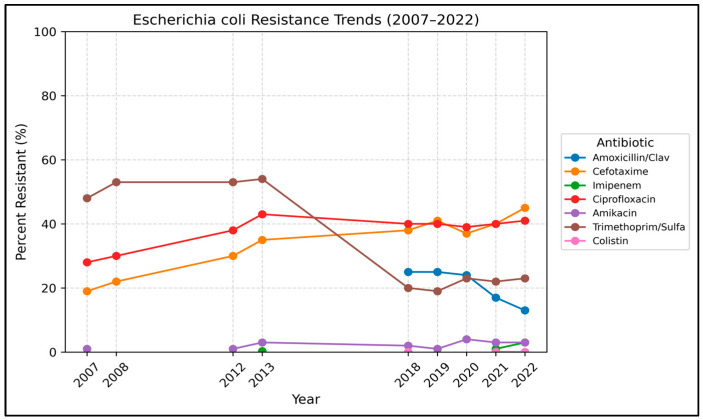
Annual resistance trends in *Escherichia coli* (2007–2022). Percent resistant over time for key antibiotics. Dashed segments indicate years with missing data.

Resistance trends for *E. coli* are presented in Table 3 and Figure 3. A total of 17,173 non-duplicate *E. coli* isolates were reported and showed resistance to several broad-spectrum antibiotics, which further increased over time. Resistance to third-generation cephalosporins increased significantly: cefotaxime and ceftazidime resistance increased from 19% to 45% and 19% to 40%, respectively, from 2007 to 2022 (OR 1.06 per year for each, *p* < 0.001). In addition, *E. coli* resistance to ciprofloxacin increased from 28% in 2007 to 41% in 2022 (OR 1.03, *p* < 0.001).

On the other hand, *E. coli* showed a significant decrease in resistance to several first-line antibiotics. Trimethoprim-sulfamethoxazole (TMP-SMX) resistance showed a decreased trend from 48% in 2007 to 23% in 2022 (OR 0.89 per year, *p* < 0.001). Similarly, nitrofurantoin and amoxicillin-clavulanate showed a downward trend from 11% in 2007 to 5% by 2022 and from 25% in 2018 to 13% in 2022 (*p* < 0.001), respectively (tested 2018 to 2022 only).

### 2.5. Antimicrobial Resistance Trends in Pseudomonas aeruginosa

**Figure 4:**
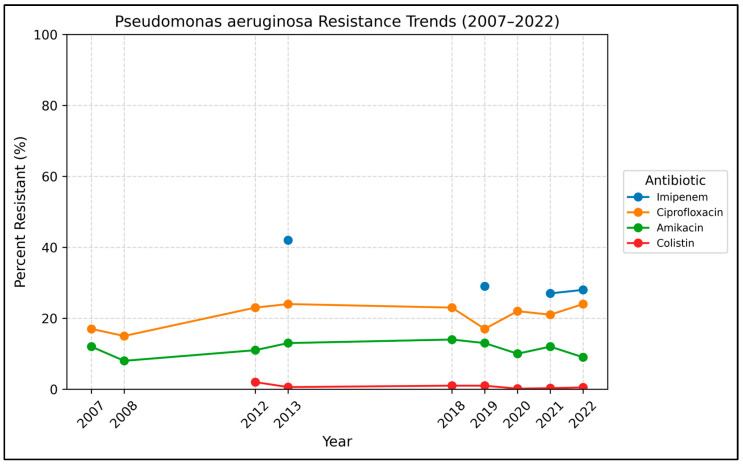
Annual resistance trends in *Pseudomonas aeruginosa* (2007–2022). Percent resistant over time for key antibiotics. Dashed segments indicate years with missing data.

Resistance trends for *Pseudomonas aeruginosa* are presented in Table 4 and Figure 4. A total of 5643 non-duplicate *P. aeruginosa* were reported.

Piperacillin-tazobactam susceptibility testing, introduced from 2018 onward, showed a slight increase in resistance from 20% in 2018 to 24% in 2022 (*p* = 0.009). Moreover, ciprofloxacin showed an increase in resistance from 17% in 2007 to 24% in 2022 (*p* = 0.026). While this trend did not meet our adjusted significance threshold (*q* > 0.05 after FDR correction within *Pseudomonas*), the minor increase in ciprofloxacin resistance aligns with global patterns and is clinically relevant.

In contrast, amikacin and gentamicin resistance declined from 12% to 9% and from 20% to 14%, respectively (*p* = 0.68 and *p* = 0.765), although these changes were not statistically significant.

Interestingly, piperacillin resistance has trended significantly downward from 24% in 2007 to 3% in 2018 (the last year tested) (OR 0.90 per year, *p* < 0.001). Similarly, imipenem resistance showed a significant downward trend from 42% in 2013 to 28% in 2022 (OR 0.93, *p* < 0.001).

### 2.6. Other Gram-Negative Organisms

Several other Gram-negative organisms have been analysed for antibiotic resistance and trends. These organisms were *Enterobacter* spp., *Citrobacter* spp., *Proteus* spp., *Haemophilus influenzae*, and *Salmonella* spp. In general, fewer isolates of these species have been reported over the years, and antibiotic testing varied across the years. Detailed data and results are attached in Supplementary Tables S1–S4 and Supplementary Figure S1.

- *Enterobacter* spp.: As shown in Table S1, there was a non-significant increase in resistance to amikacin, gentamicin, cefotaxime, and ceftazidime. However, resistance to imipenem emerged in 2018, increasing significantly from 0% to 2%.
- *Citrobacter* spp.: As shown in Table S2, resistance to nitrofurantoin showed a significant downward trend (*p* ≤ 0.001) (aligns with its activity in urinary isolates). Similarly, gentamicin showed a significant downward trend from 18 to 7% (*p* ≤ 0.001). In contrast, resistance to ceftazidime increased with time (13% to 23%), yet this trend did not reach statistical significance.
- *Proteus* spp.: Table S3 showed increased resistance to ciprofloxacin, cefotaxime, ceftazidime, and gentamicin. But these changes were not statistically significant.
- *Haemophilus influenzae*: The data available from 2018 onward showed increased resistance to cefuroxime and amoxicillin-clavulanate (2018 to 2022). Yet these trends did not reach statistical significance.
- *Salmonella* spp.: As shown in Table S4, *Salmonella* spp. showed low resistance to cefotaxime with no significant increase over time. However, resistance to ciprofloxacin increased from 29 to 49%, though this change was not statistically significant.

## 3. Discussion

This 16-year surveillance study of Gram-negative pathogenic bacteria at Mubarak Al-Kabeer Hospital showed clear evidence of increasing antibiotic resistance (AMR) in major pathogens with an important effect on patient health. *E. coli*, which is the most prevalent isolate, showed a significant increase in resistance to cefotaxime, ceftazidime, and ciprofloxacin, from 2007 to 2022, *K. pneumoniae* showed high resistance, especially the sharp increase in resistance to amikacin, meropenem and imipenem (approximately one third of isolates by 2022), in addition to high resistance to cefotaxime. In this study, *Acinetobacter* spp. were highly antibiotic-resistant, with over 75% of isolates non-susceptible to almost all tested antibiotics; only colistin showed activity (<2% resistance). In contrast, *P. aeruginosa* showed a more stable resistance trend, with a slight increase in imipenem non-susceptibility over time, and susceptibility to most other anti-pseudomonal antibiotics remained largely unchanged. Overall, these results highlighted the persistence of drug resistant Gram-negative pathogens in Mubarak Al-Kabeer Hospital. This aligns with global concerns, as the WHO has classified carbapenem-resistant *A. baumannii*, carbapenem-resistant *P. aeruginosa*, and carbapenem-resistant (ESBL producers) *Enterobacterales* as top-priority pathogens for the development of new antibiotics [17].

*Escherichia coli* was the most frequently isolated pathogen, consistent with previous regional and global reports, likely due to its role as a common commensal organism and its association with both community- and healthcare-associated infections [14,18]. The observed increase in resistance to cephalosporins and ciprofloxacin may reflect widespread antibiotic use and the spread of resistant strains, including ESBL-producing organisms [18]. Similar factors may explain the increasing resistance trends in *Klebsiella pneumoniae*, particularly in hospital settings [19,20,21,22]. These findings have important public health implications. The increasing resistance to commonly used antibiotics may limit treatment options, lead to prolonged hospital stays, and increase healthcare costs. In particular, rising resistance in *Klebsiella pneumoniae* and *Acinetobacter baumannii* is concerning due to their association with healthcare-associated infections. These trends highlight the urgent need for strengthened antibiotic stewardship programs, continuous surveillance, and infection control measures at both hospital and national levels [23]. This study’s results align with reports from Gulf countries, the Middle East region, and globally [18,19,20,21,22,23]. *E. coli* and *K. pneumoniae* are among the most common causes of Gram-negative infections in hospitals in the Middle East, and high rates of resistance have been documented [14,18,19]. A recent review reported that in some Middle Eastern hospitals (including Gulf countries), Enterobacterales resistance to carbapenems and multidrug resistance (MDR) reached alarming levels, with 100% resistance rates in certain settings [19]. Surveillance data from a global scale showed a similar pattern of increased resistance. In the last decade, specifically in 2014, around 41% of *E. coli* isolates in some national databases were already resistant to third-generation cephalosporins and fluoroquinolones, as reported by WHO [24]. Similarly, 48% of *K. pneumoniae* isolates were non-susceptible to third-generation cephalosporins, and approximately 54% were carbapenem-resistant in several WHO regions, particularly in the South-East Asia and Eastern Mediterranean Regions [24].

Our results on the rise of cefotaxime and ciprofloxacin resistance in *E. coli* align with worldwide reports on extended-spectrum β-lactamase (ESBL) producers and quinolone-resistant species [25]. In the meantime, the prevalence of meropenem and imipenem -resistant *Klebsiella* in the study hospital (up to 35%) is in line with the regional spread of carbapenemase-producing *K. pneumoniae*. For instance, reports from Iran demonstrate that over 50% of *K. pneumoniae* isolates are resistant to carbapenems [20], and hospital outbreaks in Saudi Arabia and other Gulf countries documented resistance up to 100% [21]. Our findings suggest an increasing trend in carbapenem-resistant *Klebsiella pneumoniae* in the region [22]. This pathogen is top listed globally as AMR threat because it is common and highly antibiotic resistant [22]. In comparison, our results showed that *E. coli* showed low resistance to amikacin and piperacillin-tazobactam. This suggests that these antibiotics remain useful for *E. coli* infections. In addition, several international studies [20,21,22] demonstrate that amikacin retains activity against *Enterobacterales*. Careful use of such antibiotics could help preserve their efficacy.

*Acinetobacter baumannii* is well recognized as a multidrug-resistant pathogen associated with healthcare-associated infections and limited treatment options [26]. In this study, several antibiotic classes, including cephalosporins, quinolones, and aminoglycosides, showed very limited activity, whereas colistin remained largely effective against this pathogen. Similar results have been reported from Saudi Arabia, where *A. baumannii* resistance exceeded 50% to all antibiotics except gentamicin and colistin [27]. Nationwide surveillance in Oman showed that *A. baumannii* isolates are resistant to major drug classes [28]. In addition, carbapenem-resistant *A. baumannii* have been reported from Jordan, Lebanon, and Iraq with resistance to major antibiotics above 80% [21,29]. In our study, *A. baumannii* is resistant to major antibiotics (meropenem, imipenem and ciprofloxacin). Over time, the resistance rate to amikacin, piperacillin-tazobactam, tigecycline, and cefotaxime is over 75%, which is in line with regional reports. Yet *Acinetobacter* susceptibility to colistin in our study remains very low (2%) compared with reports from the Gulf and globally [30]. Although colistin remains a last-resort antimicrobial agent for this pathogen, cautious stewardship of colistin is required, as some reports have shown that colistin-resistant *A. baumannii* (mediated by *mcr* plasmid genes or LPS modification) have emerged elsewhere [31]. It is worth mentioning that a recent report from the United Arab Emirates (UAE) showed a significant decline in *Acinetobacter* resistance rates from 2010 to 2021, with nationwide carbapenem-resistant *A. baumannii* rates falling below 30% [12]. These low figures may reflect the impact of effective infection control measures and antimicrobial stewardship programs. Similar patterns of antimicrobial resistance have also been reported in regional studies [32]. Evidence from the UAE further emphasizes that improvement is possible and highlights the importance of continued interventions [33]. However, in most of the Middle Eastern countries, including Kuwait *A. baumannii* remains a challenge align with its classification as top priority pathogen by WHO [34].

In contrast, in our study, *P. aeruginosa* has not yet developed resistance to the same degree. A slight increase in *P. aeruginosa* resistance was observed over the 16-year period, while the majority of isolates remained susceptible to key anti-pseudomonal antibiotics. A review of *P. aeruginosa* in the Arabian Gulf (2010 to 2021) documented that meropenem resistance ranged from 10% to 46% across Gulf countries, and colistin resistance remained low (0 to 0.8%) [35]. In our study, *Pseudomonas* resistance rates are within the Gulf range. It is worth mentioning that the same review documented a slight increase in certain resistances with time (12 to 17% increase in *P. aeruginosa* non-susceptibility to carbapenem in Saudi Arabia) [35], which aligns with the slight increase in resistance observed. Worldwide, carbapenem-resistant *P. aeruginosa* is recognized as a health threat, although its prevalence varies by region. In European networks, for example, carbapenem-resistant *P. aeruginosa* rates remained around 20 to 30% [36], while higher resistance levels have been reported in several Asian hospitals and healthcare settings [37]. Overall, *P. aeruginosa*’s ability for resistance (via efflux pumps, enzyme production, and mutation) means it could quickly worsen if selective pressure increases [38]. Even if the carbapenem-resistant *P. aeruginosa* resistance rate is lower than that of the other pathogens mentioned earlier, continued caution is necessary. In addition, the WHO’s classification of this pathogen as a top priority underscores the need for new antibiotics and control strategies to prevent it from becoming a superbug.

Antimicrobial stewardship and surveillance: This study highlights the need for urgent antibiotic stewardship programs and continuous surveillance at both hospital and national levels. The increase in resistance to antibiotics such as cephalosporins and quinolones may be influenced by antimicrobial use patterns. In many low- and middle-income countries, a high level of antibiotic consumption is listed in the WHO watch category of antibiotics (including most broad-spectrum antibiotics) compared to the narrow-spectrum Access category [23]. This behavior has been observed in the Eastern Mediterranean region and is strongly associated with an increase in AMR cases [23]. Therefore, the main stewardship priority is to limit the inappropriate use of the WHO watch-list antibiotics. For example, avoiding empirical quinolones for mild infections or non-controlled use of piperacillin-tazobactam and carbapenem in Intensive Care Units (ICU). The example of the UAE case suggests that antibiotic stewardship surveillance in combination with an infection control plan can achieve a real improvement [33]. Continuous surveillance is a very important step for stewardship and policy making plans. This study highlights the need to establish a broader surveillance network in Kuwait that incorporates data from multiple hospitals. These findings should be confirmed and expanded through a national AMR surveillance program, similar to those implemented in neighboring countries.

Strengths and limitations: This study has several strengths. It includes a large number of isolates (39,200) collected over a long period (16 years), representing one of the most comprehensive longitudinal assessments of antimicrobial resistance (AMR) in Kuwait. In addition, it provides a valuable long-term overview of local AMR trends, which can support empiric therapy decisions and help detect emerging resistance threats. The long-term surveillance time allowed us to use statistical trend analysis to distinguish true temporal changes from random variability. This study presents resistance data for multiple important antibiotic classes and various Gram-negative pathogens, providing a comprehensive overview of resistance in this hospital. However, several important limitations should be considered. First, as a single-center retrospective study, the findings may not be fully generalizable to other healthcare settings in Kuwait. However, the large number of isolates and long study period provide valuable insight into local AMR trends. Multi-center surveillance would strengthen national data but requires integration of laboratory information systems across hospitals. Furthermore, the analysis was not stratified by infection type, patient setting, or specimen source, and data on antimicrobial consumption (e.g., DDD and DHD) were not available. These limitations restrict the interpretation of resistance trends and their relationship to antibiotic use. In addition, data were not available for certain years within the study period due to limitations in data retrieval from the laboratory information system, which may affect the continuity of trend analysis.

Second, changes in antimicrobial susceptibility testing over the 16-year period, including updates in CLSI breakpoints and potential modifications in testing methods, may have influenced the classification of isolates as susceptible, intermediate, or resistant. Although efforts were made to apply consistent interpretation criteria, these variations may have affected the comparability of resistance trends over time.

Third, this study focused on phenotypic antimicrobial susceptibility patterns and did not investigate underlying resistance genes or molecular mechanisms. Future studies incorporating molecular analyses are needed to better understand the genetic basis of observed resistance trends. In addition, multidrug-resistant (MDR) and extensively drug-resistant (XDR) phenotypes were not specifically analyzed in this study, which should be considered in future investigations.

Even with these limitations, this study provides a valuable long-term perspective on AMR in Kuwait. The observed increases in resistance, particularly among *Klebsiella* and *Acinetobacter*, highlight the need for strengthened antibiotic stewardship and infection control. Continued surveillance and further research are essential to guide empirical therapy and evaluate intervention strategies.

## 4. Materials and Methods

### 4.1. Setting and Study Design

This retrospective surveillance study was conducted at Mubarak Al-Kabeer Hospital, a tertiary care center in Kuwait, and included both inpatient and outpatient settings from 2007 to 2022, in accordance with the STROBE guidelines for observational studies [39]. All clinically significant Gram-negative bacterial isolates were obtained from clinical specimens. Specimen sources for the isolates include blood cultures, urine culture, culture of respiratory specimen (sputum, endotracheal aspirates, and bronchoalveolar lavage), pus and wound swabs, and other sterile body fluids (cerebrospinal, pleural, and ascitic fluids).

Based on routine laboratory criteria and clinical interpretation, the included isolates were considered clinically significant. In addition, surveillance or environmental cultures were excluded. Duplicate isolates for patients with multiple isolates were excluded to ensure each strain was counted once. Any repeated isolate of the same species from the same patient and specimen site within a 30-day interval was considered duplicate, and only the first isolate was included. A total of 39,200 non-duplicate clinically significant Gram-negative bacteria were reported and analyzed in this study after duplicates were removed. This retrospective study design is appropriate for evaluating long-term antimicrobial resistance trends using routinely collected laboratory data. However, as an observational and laboratory-based study, it is primarily descriptive and does not allow for causal inference or detailed clinical stratification. Therefore, the findings should be interpreted within the context of these inherent limitations.

### 4.2. Bacterial Isolates and Antimicrobial Susceptibility Testing

All isolates were identified to the species level using standard microbiological procedures including automated identification systems (VITEK 2 Compact, bioMérieux, Marcy L’Etoile, France) and Matrix-Assisted Laser Desorption/Ionization—Time of Flight Mass Spectrometry (MALDI-TOF MS) (VITEK MS, bioMérieux, Marcy l’Etoile, France). Antibiotic susceptibility testing (AST) was conducted according to the hospital’s standard procedures, including broth microdilution for minimum inhibitory concentration (MIC) testing, as per the quality control procedures. Antimicrobial susceptibility results were interpreted according to CLSI M100 guidelines [40,41]. Where applicable, CLSI M45 [42] recommendations were used for fastidious organisms such as *Haemophilus influenzae*. Colistin susceptibility was determined by agar dilution according to CLSI guidelines [40,41]. Results interpretation for susceptibility based on the Clinical and Laboratory Standard Institute (CLSI) guidelines according to the standard update to each year of the study. AST results were categorized as susceptible, intermediate, or resistant according to the CLSI breakpoint tables (M100 standards) at the time of testing [40,41].

Intermediate results were classified as resistant to yield a binary outcome (susceptible vs. non-susceptible), consistent with approaches commonly used in antimicrobial resistance surveillance studies to facilitate trend analysis. However, this approach may lead to a slight overestimation of resistance rates, and results should be interpreted with this consideration. All antibiotics included in this study were routinely tested for the reported species in the clinical laboratory during the study period.

### 4.3. Data Management and Definitions

Data for each isolate (species identification, and AST results) were obtained from the laboratory information system (LIS) and entered into a de-identified database. All clinically significant Gram-negative isolates collected from inpatient and outpatient specimens during the study period were included. Only non-duplicate isolates were considered. Environmental isolates, surveillance cultures, and likely contaminants were excluded from the analysis.

Due to the retrospective design and limitations of the LIS, analyses were not stratified by infection type, patient setting, or specimen source, and antimicrobial consumption data (e.g., DDD/DHD) were not available for inclusion in the analysis. In addition, patient-level demographic and clinical data (e.g., age, sex, comorbidities, prior infection history, and immune status) were not available in the laboratory information system and were therefore not included in the analysis. Each isolate was tested against multiple antimicrobial agents; therefore, the number of observations varies by antibiotic, as not all isolates were tested against all agents. Antimicrobial susceptibility testing was not repeated for the same isolation. Accordingly, the unit of analysis in this study was the isolate–antibiotic test result. The database was reviewed to remove datasets that did not meet the study criteria (environmental isolates and contaminants) and to merge duplicate entries. AST results for each isolate were tabulated by antibiotic; therefore, the unit of analysis was isolate-antibiotic test result (each group of isolate-antibiotic results was treated as one set for statistical analysis). Isolates categorized as either resistant or intermediate were classified as non-susceptible for the purpose of analysis. This study did not focus on multidrug-resistant species and extremely drug-resistant bacteria; therefore, these were not reported, yet overall resistance patterns were described. Therefore, the findings of this study should be interpreted as descriptive resistance patterns rather than as explanations of underlying clinical or epidemiological factors.

### 4.4. Ethical Considerations

This study used anonymized, retrospective laboratory data with no direct patient identifiers. Ethical approval was obtained from the ethics committee of Ministry of Health, Kuwait (approval number 1604/2024). Given the nature of the data, the requirement for informed consent was waived.

### 4.5. Statistical Analysis

Annual resistance rates (percentage of isolates non-susceptible each year) were analyzed for temporal trends. In this study, we used logistic regression to assess trends in resistance over time, treating the isolate collection year as a continuous independent variable (predictor) and the susceptibility results (resistant vs. susceptible) as the dependent variable for each organism-antibiotic group. This analysis used an odds ratio (OR) for the change in odds of resistance per year along with its 95% confidence interval (CI) [38]. For instance, in the case of *K. pneumoniae* and meropenem, the OR per year showed a multiplicative increase in odds of meropenem resistance with each year. In addition, OR > 1 signifies an increasing trend in resistance, while OR < 1 shows a downward trend. The significance of each separate trend was evaluated by the Wald chi-square test on the year coefficient, and a *p*-value for trend was obtained for each organism-antibiotic group [43,44].

Given the large number of resistance trends analyzed, multiple-comparison adjustment was performed using the Benjamini–Hochberg false discovery rate (FDR) to control the type I error rate. The *p*-values from the logistic regression trend tests were adjusted to yield *q*-values, and an FDR threshold of 0.05 was used to determine statistical significance. The asterisk (*) in the reported tables was used to denote trends that stay significant after FDR adjustment (*q* < 0.05) [45]. All analyses in the study were two-tailed and performed using statistical software (SPSS Statistics version 28.0 and R version 4.2) [46,47]. Our results were presented with ORs, 95% Cis, and *p*-values for annual trends. All trends for the tested organisms were reported in detail in the results section, and tables and figures were provided to demonstrate long-term changes in antibiotic susceptibility between 2007 and 2022.

## 5. Conclusions

This study provides a long-term overview of antimicrobial resistance trends among major Gram-negative pathogens at a tertiary care hospital in Kuwait. Increasing resistance was observed in *E. coli* and *K. pneumoniae*, while *A. baumannii* remained highly resistant and *Pseudomonas aeruginosa* showed relatively stable patterns over time.

These findings highlight the ongoing presence and evolution of antimicrobial resistance during the study period; however, they should be interpreted with caution given the lack of stratified clinical data and antimicrobial consumption information. Further studies incorporating detailed clinical data and multi-center surveillance are needed to better understand resistance patterns and support future strategies.

## Figures and Tables

**Table 1:** Temporal trends in antimicrobial resistance in *Klebsiella pneumoniae*, 2007–2022.

Antibiotic	Years	Total n	%R (First)	%R (Last)	OR/Year (95% CI)	*p*-Value	*q*-Value (FDR)
Cefotaxime	2007–2022	15,057	27%	60%	1.106 (1.098–1.114)	<0.001	<0.001
Ceftazidime	2007–2022	14,905	27%	58%	1.109 (1.101–1.117)	<0.001	<0.001
Cefuroxime	2007–2022	14,381	29%	64%	1.110 (1.103–1.118)	<0.001	<0.001
Cefoxitin	2018–2022	13,716	12%	45%	1.594 (1.512–1.679)	<0.001	<0.001
Meropenem	2008–2022	23,967	1%	35%	1.622 (1.560–1.686)	<0.001	<0.001
Piperacillin	2007–2021	12,484	52%	42%	0.971 (0.958–0.984)	<0.001	<0.001
Ampicillin **	2007–2022	8439	100%	100%	–	–	–
Piperacillin–Tazobactam	2018–2022	8147	36%	40%	1.034 (1.004–1.066)	0.029	0.04
Amoxicillin–Clavulanate	2007–2013	2635	34%	34%	0.990 (0.949–1.033)	0.378	0.45
Amikacin	2007–2022	8439	2%	22%	1.251 (1.216–1.287)	<0.001	<0.001
Gentamicin	2007–2022	8439	7%	16%	1.055 (1.016–1.096)	0.009	0.02
Ciprofloxacin	2007–2022	8439	22%	49%	1.106 (1.099–1.113)	<0.001	<0.001
Trimethoprim–Sulfamethoxazole	2007–2022	8439	30%	28%	0.987 (0.974–1.001)	0.072	0.09
Nitrofurantoin	2007–2022	3074	75%	40%	0.858 (0.842–0.874)	<0.001	<0.001
Colistin	2012–2022	6278	0.0%	0.3%	1.055 (0.669–1.667)	0.866	0.90

** indicates intrinsic resistance (shown for completeness; temporal trend not modelled or interpreted). Total n = number of *Klebsiella pneumoniae* isolates tested for each antimicrobial agent. %R (first) = percentage resistance in the first year; %R (last) = percentage resistance in the last year. OR/year (95% CI) = odds ratio per year (95% confidence interval). *p*-value = *p*-value for trend; *q*-value (FDR) = *p*-value adjusted for multiple comparisons using the false discovery rate. Total n may differ from the total number of isolates, as not all isolates were tested against all antimicrobial agents.

**Table 2:** Temporal trends in antimicrobial resistance among *Acinetobacter baumannii* (2007–2022).

Antibiotic	Years	Total n	%R (First)	%R (Last)	OR/Year (95% CI)	*p*-Value	*q*-Value (FDR)
Cefotaxime	2007–2013	1133	89%	95%	1.100 (0.996–1.214)	0.060	0.08
Meropenem	2007–2022	3557	33%	77%	1.071 (1.053–1.089)	<0.001	<0.001 *
Piperacillin–Tazobactam	2018–2022	2424	5%	78%	2.307 (2.128–2.501)	<0.001	<0.001 *
Piperacillin	2007–2018	1684	59%	7%	0.691 (0.665–0.717)	<0.001	<0.001 *
Amikacin	2007–2022	3557	33%	51%	0.991 (0.976–1.006)	0.260	0.30
Gentamicin	2007–2022	3557	42%	37%	0.904 (0.890–0.918)	<0.001	<0.001 *
Ciprofloxacin	2007–2022	3557	54%	77%	1.008 (0.990–1.026)	0.396	0.45
Trimethoprim–Sulfamethoxazole	2007–2022	3557	50%	47%	0.898 (0.884–0.913)	<0.001	<0.001 *
Colistin	2013–2022	2340	0.3%	2.0%	1.246 (1.046–1.485)	0.014	0.03 *

Total n = number of *Acinetobacter baumannii* isolates tested for each agent, %R (first) = percent of resistance in the first year, %R (last) = percent of resistance in recent years, OR/year (95% CI) = Odds Ratio per year (with 95% Confidence Interval), *p*-value = the *p*-value for trend significance and * indicates *p* < 0.05. Total n represents the number of isolates tested for each antimicrobial agent and may differ from the total number of isolates, as not all isolates were tested against all antibiotics.

**Table 3:** Temporal trends in antimicrobial resistance among *Escherichia coli* isolates (2007–2022).

Antibiotic	Years	Total n	%R (First)	%R (Last)	OR/Year (95% CI)	*p*-Value	*q*-Value (FDR)
Cefotaxime	2007–2022	24,157	19%	45%	1.072 (1.063–1.081)	<0.001	<0.001
Cefuroxime	2007–2022	24,787	25%	51%	1.064 (1.056–1.072)	<0.001	<0.001
Ceftazidime	2007–2022	25,314	19%	40%	1.065 (1.057–1.073)	<0.001	<0.001
Cefoxitin	2007–2022	2123	8%	13%	1.134 (1.075–1.195)	<0.001	<0.001
Cephalothin	2007–2013	6571	58%	63%	1.035 (1.016–1.054)	<0.001	<0.001
Imipenem	2013–2022	1491	0.3%	3%	1.320 (1.178–1.479)	<0.001	<0.001
Meropenem	2007–2022	24,157	0.2%	3%	1.016 (0.969–1.065)	0.398	0.45
Ampicillin	2007–2022	15,408	73%	79%	1.003 (0.976–1.006)	0.339	0.40
Piperacillin	2007–2021	12,484	52%	51%	0.971 (0.958–0.984)	<0.001	<0.001
Piperacillin–Tazobactam	2007–2022	5687	2–15%	4–14%	–	–	–
Amoxicillin–Clavulanate	2018–2022	9109	25%	13%	0.818 (0.806–0.831)	<0.001	<0.001
Amikacin	2007–2022	3557	1%	3%	1.071 (1.034–1.109)	<0.001	<0.001
Gentamicin	2007–2022	17,173	16%	14%	0.951 (0.938–0.964)	<0.001	<0.001
Ciprofloxacin	2007–2022	17,173	28%	41%	1.031 (1.025–1.037)	<0.001	<0.001
Norfloxacin	2007–2013	6089	27%	34%	1.063 (1.044–1.083)	<0.001	<0.001
Trimethoprim–Sulfamethoxazole	2007–2022	17,173	48%	23%	0.893 (0.884–0.903)	<0.001	<0.001
Nitrofurantoin	2007–2022	17,173	11%	5%	0.927 (0.903–0.952)	<0.001	<0.001
Tigecycline	2010–2022	3542	0.9%	0.05%	0.755 (0.649–0.879)	<0.001	<0.001
Colistin	2013–2022	1857	0.05%	0.05%	1.055 (0.669–1.667)	0.866	0.90

Total n = number of *Escherichia coli* isolates tested for each antimicrobial agent. %R (first) = percentage resistance in the first year; %R (last) = percentage resistance in the last year. OR/year (95% CI) = odds ratio per year (95% confidence interval). *p*-value = *p*-value for trend; *q*-value (FDR) = *p*-value adjusted for multiple comparisons using the false discovery rate. Total n may differ from the total number of isolates, as not all isolates were tested against all antimicrobial agents.

**Table 4:** Temporal trends in antimicrobial resistance among *P. aeruginosa*, 2007–2022.

Antibiotic	Years	Total n	%R (First)	%R (Last)	OR/Year (95% CI)	*p*-Value	*q*-Value (FDR)
Ceftazidime	2007–2022	5643	34%	29%	0.978 (0.967–0.989)	<0.001	<0.001 *
Imipenem	2013–2022	2713	42%	28%	0.926 (0.905–0.948)	<0.001	<0.001 *
Piperacillin–Tazobactam	2018–2022	3420	20%	24%	1.081 (1.020–1.145)	0.009	0.02 *
Piperacillin	2007–2018	2902	24%	3%	0.896 (0.874–0.918)	<0.001	<0.001 *
Amikacin	2007–2022	5643	12%	9%	1.003 (0.987–1.020)	0.680	0.72
Gentamicin	2007–2022	5643	20%	14%	0.998 (0.984–1.012)	0.765	0.80
Ciprofloxacin	2007–2022	5643	17%	24%	1.015 (1.002–1.028)	0.026	0.05 *
Colistin	2012–2022	4757	2.0%	0.5%	0.881 (0.809–0.958)	0.003	0.01 *

Total n = number of *Pseudomonas aeruginosa* isolates tested for each antimicrobial agent. %R (first) = percentage resistance in the first year; %R (last) = percentage resistance in the last year. OR/year (95% CI) = odds ratio per year (95% confidence interval). *p*-value = *p*-value for trend; *q*-value (FDR) = *p*-value adjusted for multiple comparisons using the false discovery rate. * indicates statistically significant trend after FDR correction (*q* < 0.05). Total n may differ from the total number of isolates, as not all isolates were tested against all antimicrobial agents.

## Data Availability

The data supporting this study is not publicly available due to institutional privacy policies and ethical restrictions. The anonymized laboratory dataset is held by Mubarak Al-Kabeer Hospital, Ministry of Health and can be accessed only with permission from the Ministry of Health’s ethics committee.

## Supplementary Materials

The following supporting information can be downloaded at: https://www.mdpi.com/article/10.3390/antibiotics15050501/s1, Table S1: Trends in antimicrobial resistance among *Enterobacter* spp. isolates, 2007–2022; Table S2: Trends in antimicrobial resistance among *Citrobacter* spp. isolates, 2007–2022; Table S3: Trends in antimicrobial resistance among *Proteus* spp. isolates, 2007–2022; Table S4: Trends in antimicrobial resistance among *Salmonella* spp. isolates, 2007–2022; Figure S1: Resistance trends for the “other Gram-negative” species (*Enterobacter* spp., *Proteus* spp., *Citrobacter* spp., and *Salmonella* spp., 2007–2022).

## Abbreviations

The following abbreviations are used in this manuscript:

AMR	Antimicrobial Resistance
Amoxicillin–Clav	Amoxicillin–Clavulanate
AST	Antimicrobial Susceptibility Testing
CI	Confidence Interval
CLSI	Clinical and Laboratory Standards Institute
FDR	False Discovery Rate
GLASS	Global Antimicrobial Resistance Surveillance System
IRB	Institutional Review Board
LIS	Laboratory Information System
MALDI-TOF- MS	Matrix-Assisted Laser Desorption/Ionization–Time of Flight Mass Spectrometry
MDR	Multidrug-Resistant
MIC	Minimum Inhibitory Concentration
OR	Odds Ratio
Piperacillin–Tazo	Piperacillin–Tazobactam
TMP SMX	Trimethoprim–Sulfamethoxazole
Trimethoprim-Sulfa	Trimethoprim–Sulfamethoxazole
WHO	World Health Organization
XDR	Extensively Drug-Resistant
