# Supplementary material for: Long-Term Trends in Antimicrobial Resistance Among Gram-Negative Clinical Isolates at Mubarak Al-Kabeer Hospital, Kuwait (2007–2022)
Source: Antibiotics (Basel). 2026 May 17;15(5):501. doi: 10.3390/antibiotics15050501 (PMC13203850; doi:10.3390/antibiotics15050501)
Supplement: Supplementary file 1 [file antibiotics-15-00501-s001.zip › antibiotics-4251644-supplementary.pdf]

Table S1. Trends in antimicrobial resistance among *Enterobacter* spp. isolates, 2007–2022.

Antibiotic	Years tested	Total n	%R first year	%R last year	OR per year (95% CI)	*p* value	*q* (FDR)	*
Cefotaxime	2007–2022	1233	31%	40%	1.025 (1.002–1.048)	0.036	0.113	
Ceftazidime	2007–2022	1233	32%	34%	0.997 (0.978–1.017)	0.798	0.997	
Cefuroxime	2007–2022	1233	68%	65%	0.984 (0.961–1.007)	0.179	0.500	
Cefoxitin	2007–2022	1233	12%	25%	1.050 (1.028–1.072)	<0.001	<0.001	*
Cephalothin	2007–2013	302	45%	25%	0.765 (0.625–0.937)	<0.001	0.000	*
Imipenem	2013–2022	557	0%	2%	0.670 (0.512–0.876)	<0.001	<0.001	*
Meropenem	2007–2022	1233	0%	0%	1.030 (0.970–1.089)	0.170	0.500	
Ampicillin	2007–2022	1233	99%	100%	–	–	–	
Piperacillin	2007–2021	1081	35%	33%	1.023 (0.998–1.048)	0.069	0.153	
Piperacillin–Tazobactam	2007–2022	1233	26%–50%	7%–36%	~0.20–0.88	<0.05	<0.05	*
Amoxicillin–Clavulanate	2007–2022	1117	76%–99%	45%–92%	~0.67–0.78	<0.05	<0.05	*
Amikacin	2007–2022	1233	4%	6%	1.001 (0.889–1.127)	0.988	0.999	
Gentamicin	2007–2022	756	4%	6%	1.001 (0.798–1.255)	0.986	0.999	
Ciprofloxacin	2007–2022	1233	12%	11%	0.990 (0.963–1.017)	0.441	0.766	
Norfloxacin	2007–2013	539	14%	12%	0.890 (0.786–1.009)	0.069	0.153	
Nitrofurantoin	2007–2022	1233	81%	20%	0.784 (0.762–0.806)	<0.001	<0.001	*
Tigecycline	2013–2022	810	66%	0.7%	0.714 (0.671–0.759)	<0.001	<0.001	*

‡ Indicates results reported as a range due to non-continuous testing or separate sub-period analyses. Total n = number of *Enterobacter* isolates tested for each antimicrobial agent. %R (first) = percentage resistance in the first year; %R (last) = percentage resistance in the last year. OR/year (95% CI) = odds ratio per year (95% confidence interval). p-value = p-value for trend; q-value (FDR) = p-value adjusted for multiple comparisons using the false discovery rate (Benjamini–Hochberg). * indicates statistically significant trend after FDR correction ($q < 0.05$). Total n may differ from the total number of isolates, as not all isolates were tested against all antimicrobial agents.

Table S2. Trends in antimicrobial resistance among *Citrobacter* spp. isolates, 2007–2022.

Antibiotic	Years tested	Total n	%R first year	%R last year	OR per year (95% CI)	*p* value	*q* (FDR)	*
Cefotaxime	2007–2022	533	13%	23%	1.026 (1.000–1.052)	0.048	0.317	
Ceftazidime	2007–2022	533	13%	23%	1.013 (0.984–1.043)	0.388	0.776	
Cefuroxime	2007–2022	533	23%	40%	1.018 (0.995–1.041)	0.125	0.525	
Cefoxitin	2007–2022	533	16%	30%	1.276 (1.102–1.478)	0.005	0.022	*
Cephalothin	2007–2013	152	64%	25%	0.765 (0.663–0.884)	<0.001	<0.001	*
Imipenem	2013–2022	205	0%	0%	0.916 (0.651–1.290)	0.623	0.865	
Meropenem	2007–2022	533	0%	0%	0.984 (0.930–1.041)	0.554	0.865	
Ampicillin	2007–2022	576	97%	100%	1.023 (0.969–1.080)	0.447	0.525	
Piperacillin	2007–2021	445	68%	45%	0.944 (0.909–0.981)	0.004	0.018	*
Piperacillin–Tazobactam	2008–2022	452	2%–16%	2%–11%	~0.89–1.00	0.990	1.000	
Amoxicillin–Clavulanate	2007–2022	576	28%–42%	24%–28%	~0.78–1.00	0.060	0.087	
Amikacin	2007–2022	533	3%	2%	0.945 (0.806–1.109)	0.494	0.865	
Gentamicin	2007–2022	533	18%	7%	0.860 (0.786–0.941)	<0.001	<0.001	*
Ciprofloxacin	2007–2022	533	19%	15%	0.968 (0.926–1.012)	0.150	0.525	
Norfloxacin	2007–2013	146	8%	12%	1.063 (1.016–1.112)	0.009	0.034	*
Nitrofurantoin	2007–2022	533	19%	6%	0.908 (0.851–0.969)	<0.001	<0.001	*
Tigecycline	2013–2022	221	14%	3%	0.811 (0.706–0.932)	0.003	0.017	*

‡ Indicates results reported as a range due to non-continuous testing or separate sub-period analyses. Total n = number of *Citrobacter* isolates tested for each antimicrobial agent. %R (first) = percentage resistance in the first year; %R (last) = percentage resistance in the last year. OR/year (95% CI) = odds ratio per year (95% confidence interval). p-value = p-value for trend; q-value (FDR) = p-value adjusted for multiple comparisons using the false discovery rate (Benjamini–Hochberg). * indicates statistically significant trend after FDR correction ($q < 0.05$). Total n may differ from the total number of isolates, as not all isolates were tested against all antimicrobial agents.

Table S3. Trends in antimicrobial resistance among *Proteus* spp. isolates, 2007–2022.

Antibiotic	Years tested	Total n	%R first year	%R last year	OR per year (95% CI)	*p* value	q (FDR)	*
Cefotaxime	2007–2022	1352	3%	19%	1.034 (0.999–1.070)	0.057	0.144	
Ceftazidime	2007–2022	1352	3%	14%	1.027 (0.985–1.071)	0.200	0.355	
Cefuroxime	2007–2022	1352	9%	30%	1.034 (0.994–1.075)	0.093	0.218	
Cefoxitin	2007–2022	1352	15%	30%	1.006 (0.963–1.050)	0.789	0.869	
Cephalothin	2007–2013	183	15%	30%	1.060 (1.010–1.112)	0.018	0.052	
Imipenem	2013–2022	444	0%	0%	1.037 (0.776–1.385)	0.801	0.869	
Meropenem	2007–2022	1352	0%	0%	1.025 (0.946–1.111)	0.547	0.869	
Ampicillin	2007–2022	1213	46%	52%	1.010 (0.981–1.040)	0.454	0.715	
Piperacillin	2007–2021	1111	12%	32%	0.941 (0.901–0.983)	0.006	0.020	*
Piperacillin–Tazobactam	2007–2022	546	1%–6%	1%–1%	~0.87–1.02	0.982	0.999	
Amoxicillin–Clavulanate	2007–2022	740	16%–28%	10%–28%	~0.82–1.00	<0.05	<0.05	*
Amikacin	2007–2022	1263	2%	3%	1.071 (1.034–1.109)	<0.001	<0.001	*
Gentamicin	2007–2022	894	16%	25%	1.087 (0.974–1.214)	0.135	0.280	
Ciprofloxacin	2007–2022	1352	13%	37%	1.015 (1.002–1.028)	0.026	0.065	
Norfloxacin	2007–2013	322	22%	37%	1.039 (0.999–1.080)	0.054	0.120	
Nitrofurantoin	2007–2022	1352	100%	34%	0.887 (0.864–0.911)	<0.001	<0.001	*
Tigecycline	2013–2022	276	2%	3%	1.025 (0.920–1.142)	0.639	0.869	
Colistin	2013–2022	383	0%	0%	–	–	–	

‡ Indicates results reported as a range due to non-continuous testing or separate sub-period analyses. Total n = number of *Proteus* isolates tested for each antimicrobial agent. %R (first) = percentage resistance in the first year; %R (last) = percentage resistance in the last year. OR/year (95% CI) = odds ratio per year (95% confidence interval). p-value = p-value for temporal trend; q-value (FDR) = p-value adjusted for multiple comparisons using the false discovery rate (Benjamini–Hochberg). * indicates statistically significant trend after FDR correction (q < 0.05). Total n may differ from the total number of isolates, as not all isolates were tested against all antimicrobial agents.

Table S4. Trends in antimicrobial resistance among *Salmonella* spp. isolates, 2007–2022.

Antibiotic	Years tested	Total n	%R first year	%R last year	OR per year (95% CI)	*p* value	*q* (FDR)	*
Cefotaxime	2007–2022	504	2%	8%	1.055 (0.989–1.125)	0.106	0.333	
Ampicillin	2007–2020	419	16%	3%	–	–	–	
Ciprofloxacin	2007–2022	627	29%	49%	–	–	–	
Chloramphenicol	2007–2022	627	16%	8%	0.951 (0.906–0.999)	0.045	0.181	
Trimethoprim–Sulfamethoxazole	2007–2022	627	6%	11%	0.961 (0.907–1.019)	0.186	0.334	
Tetracycline	2007–2022	627	27%	35%	0.964 (0.904–1.029)	0.194	0.387	

Total n = number of *Salmonella* isolates tested for each antimicrobial agent. %R (first) = percentage resistance in the first year; %R (last) = percentage resistance in the last year. OR/year (95% CI) = odds ratio per year (95% confidence interval). p-value = p-value for temporal trend; q-value (FDR) = p-value adjusted for multiple comparisons using the false discovery rate (Benjamini–Hochberg). * indicates statistically significant trend after FDR correction ($q < 0.05$). Total n may differ from the total number of isolates, as not all isolates were tested against all antimicrobial agents.

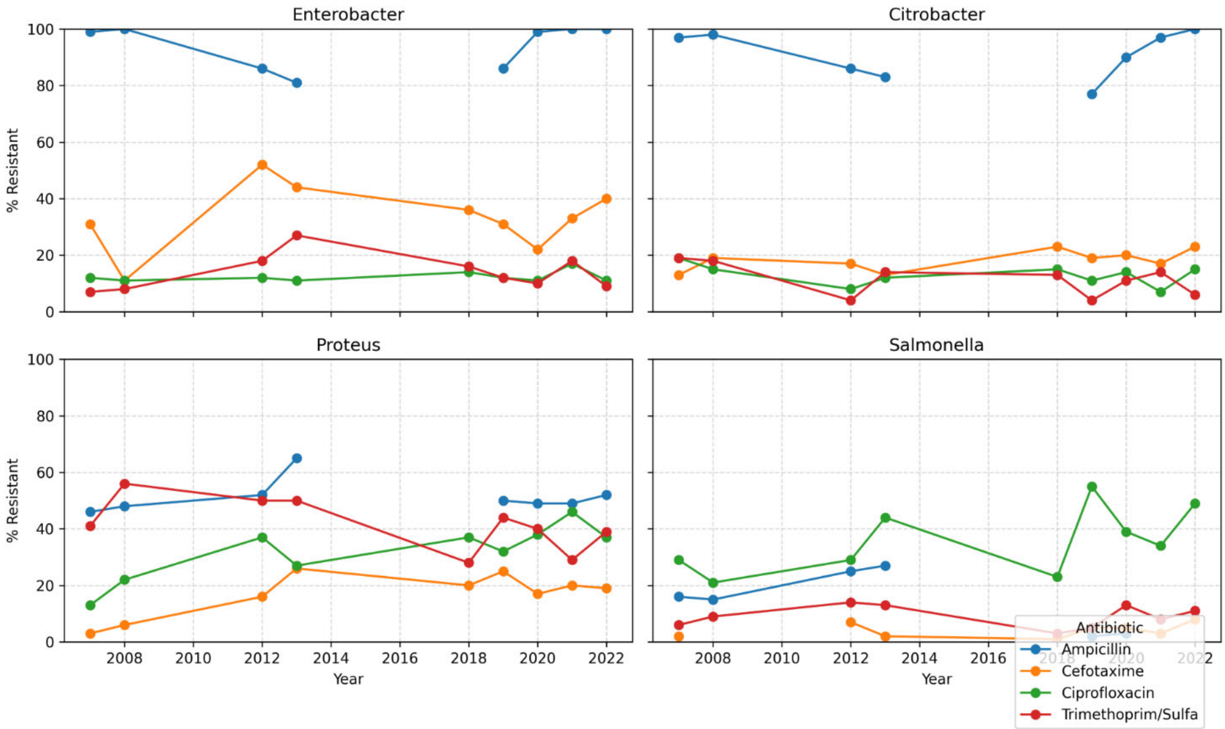

Figure S1. Resistance trends for the "other Gram-negative" species (*Enterobacter* spp., *Proteus* spp., *Citrobacter* spp., and *Salmonella* spp., 2007–2022). We show only antibiotics with interpretable data, dashed line segments mark years without testing.
